# Untangling social inequality: Psychosocial and political pathways toward health equity – A call for civic engagement

**DOI:** 10.1016/j.puhip.2026.100877

**Published:** 2026-10-27

**Authors:** Anton Matthes, Julia Nadine Doetsch

**Affiliations:** Behavioral Epidemiology, Institute of Clinical Psychology and Psychotherapy, Dresden University of Technology (TUD), Dresden, Germany

**Keywords:** Social inequality, Vulnerable individuals, Health system, Political participation, Psychosocial factors, Social determinants of health

## Abstract

**Objectives:**

To introduce the PoCID-H Model, a multilevel framework conceptualizing political participation as a fundamental determinant of health and a potential lever for reducing health inequalities in the European Union and beyond.

**Study design:**

The short communication presents a conceptual framework that integrates psychosocial mechanisms, political participation, and health outcomes within the broader context of social inequality.

**Methods:**

The short communication develops a theoretical perspective linking psychosocial mechanisms, including stress, self-efficacy, and cognition, with political participation and health outcomes. It examines the interactions between individual behaviors, policy contexts, and health systems, emphasizing cyclical feedback loops that may reinforce or mitigate social and health inequalities.

**Results:**

The PoCID-H Model highlights the interconnected relationships between psychosocial processes, political participation, and health outcomes. It underscores how social inequality may contribute to political disengagement and polarization, particularly among socioeconomically vulnerable populations, and identifies the potential role of political participation in shaping health-related policies and outcomes.

**Conclusions:**

An activist public health approach is needed to address the structural and psychosocial dimensions of health inequalities. Integrating psychosocial indicators into preventive public health practices and recognizing political participation as a fundamental determinant of health may help strengthen health equity across the European Union and beyond.

**What this study adds:**

1. Achieving Health Equity means Combining Psychosocial and Political Pathways
2. Stress, self-efficacy, cognition mediate social inequality, politics, and health
3. The PoCID-H Model links individual (micro), context (meso) and system (macro) factors

**Implications for policy and practice:**

1. Solution: Integrate psychosocial indicators in screenings via electronic patient file
2. Political participation is not civic right but fundamental determinant of health

## 1 Introduction

It is well established that social inequality remains one of the most persistent issues in determining health, shaping access to healthcare [1], and hampering equal health opportunities across generations. For example within the European Union (EU), widening inequalities have contributed to political polarization and governmental instability, often intersecting with debates on migration policies which themselves are shaped amplified by these widening inequalities [2]. In 2023, nearly six million immigrants were recorded within the EU [3]. Many of these *vulnerable* individuals – particularly immigrants and those with low socioeconomic status (SES) – experience disproportionally high social and health inequalities. Strikingly, simultaneously political participation has declined across the EU, especially among these *vulnerable* populations [4], highlighting a gap between the communities most affected by inequalities and their influence on policy decisions.

Rather than being the root cause of systemic strain, these inequalities reflect deeper social fractures and underscore the need for activism through civic engagement. This prompts three critical questions:

1. What mechanisms that arise from socioeconomic inequalities, shape individual political engagement and contemporary political trends?
2. How is low socioeconomic status (SES) associated with these trends contributing to deepening inequalities within the health system?
3. And how can the resulting social inequalities be addressed by activating civil engagement to improve equitable health governance?

## 2 Methods

### 2.1 The PoCID-H model

We argue that this disengagement in political participation [4] reflects not mere political apathy, but rather a psychosocial response to persistent structural inequalities, which end in political apathy. We highlight the role of behavior, cognition, and psychological variables of individuals explaining the response in political and health-related decision-making processes, identifying the influential relationship of social inequality (Fig. 1), building on the World Health Organization's Commission on Social Determinants of Health [5].

**Fig. 1:**
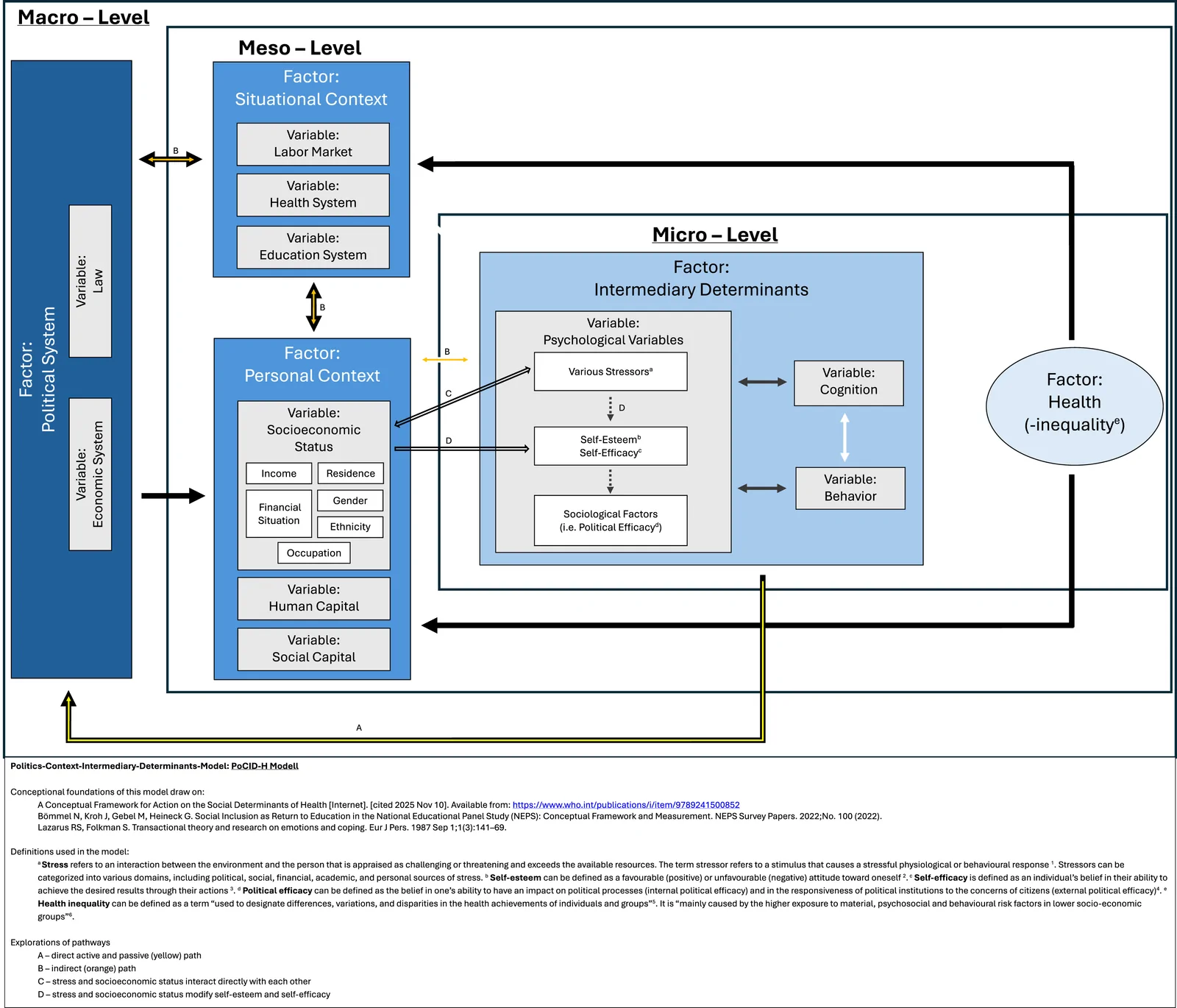
Politics-Context-Intermediary-Determinants-Model: PoCID-H Model.

The proposed PoCID-H Model depicts the role of individual-level determinants (micro-level), mediated by situational and personal contextual factors (meso-level), which might influence the political system (macro-level). Thus, it delineates a cyclical framework encompassing resultant effects, intervention and subsequent regulatory responses. While it conceptualizes this dynamic by situating psychosocial factors as mediators between structural conditions and political behavior, it also places a focus on the individual.

## 3 Results

### 3.1 Linking inequality, cognition, and political engagement

At the **micro-level**, consisting of intermediary determinants influencing the political system *direct actively* and *passively* (yellow path; A), *vulnerable* individuals (e.g. low SES) may experience higher stress leading to reduced self-esteem/self-efficacy [6], [7]. This triangular interaction (C + D) ultimately leads to alterations in political self-efficacy — shaping the perceived ability to influence political processes [4]. These changes may be reflected in cognitive beliefs and subsequently manifest in individual behavior, potentially leading to withdrawal from political engagement. Thus, individual's *passive* behavior exerts a *direct* impact on political systems, seen in declining political participation [8]. In contrast, *active direct* engagement could counteract inequality (e.g. through voting).

At **meso-level**, personal context factors constitute a central element within the PoCID-H Model. Policies enacted by the political system influencing components of the situational context exert differential effects, mediated by their socioeconomic background, on individuals. This dynamic generates advantages and disadvantages, which, e.g., in the health system, may contribute to health-related discrimination and thereby reinforces social inequality [1].

Thus, the PoCID-H Model captures feedback loops between personal context, behavior, and systemic response, linking psychosocial variables to health and political outcome.

### 3.2 The policy context: Socio-political determinants of health

The political system, **at macro-level**, regulates the situational and personal context, which influence the population in general. This *indirect* bidirectional pathway (*orange path*; B) predominantly affects groups of *vulnerable* individuals.

On the one side, policies enacted at macro-level reverberate through these individual and contextual layers, e.g. health system reforms designed without accounting for social context can *indirectly* exacerbate health inequality [1].

On the other side, vulnerable groups typically exhibit higher utilization of healthcare services and often these needs are not met, revealing systemic inequalities [1], [9]. With those groups increasing [10], political counter-regulatory action is more often required.

This creates a cyclical health system burden: as inequalities deepen, political pressure mounts for compensatory interventions, which may themselves be unevenly distributed, thereby reinforcing health inequalities.

## 4 Discussion

### 4.1 Towards activism and equitable health governance

This complex interplay demands a holistic preventative response, drawing on the HiAP/H4AP strategies. Strengthening the links between social inclusion, political participation, and equitable health systems is an **urgent public health imperative**. Hence, as a preventive measure: We call for the **integration of psychosocial indicators** — such as high perceived stress and low self-efficacy — into periodic screenings. A national example from the EU is Germany, where populations at risk of health and social inequalities could be identified through electronic patient records (**ePA**), implemented in the context of the European Health Data Space, at the **macro-level**, or through general practitioners at the **individual level.**

With individuals embedded in supportive networks, psychosocial strain and political disengagement can be mitigated. Through psychosocial-empowerment-oriented interventions, **health equity can be improved** and citizens can be **re-engaged in political processes**.

### 4.2 Strength and limitations

The main strength of this short communication is the introduction of the PoCID-H Model, which provides a multilevel conceptual perspective on the interplay between psychosocial mechanisms, political participation, and health inequalities. Although the framework is illustrated using the EU as a reference context, its potential applicability is not limited to this setting. Yet, as a conceptual contribution, the model requires further theoretical development and empirical investigation to clarify the proposed relationships and assess its implications for public health research and practice.

### 4.3 Conclusion

Bridging the gap between social inequality and health demands to **promote political participation** not merely as a civic right, but as a **fundamental determinant of health**.

## Author statements

AM: conceptualisation, data curation, formal analysis, investigation, methodology, project administration, visualisation, writing – original draft, and writing – review & editing JND: conceptualisation, data curation, formal analysis, funding acquisition, investigation, methodology, project administration, supervision, validation, visualisation, writing – original draft, and writing – review & editing.

## Ethics statement

Ethical approval was not applicable to this work because it is a commentary and did not involve human participants or human data. Accordingly, ethical approval was not obtained.

## AI statement

During the preparation of this work, the authors used ChatGPT to translate the initial draft, which was written in German, and later for minor text editing. After using this tool, the authors reviewed and edited the content as needed and take full responsibility for the content of the published article.

## Funding

The funding source “Behavioral Epidemiology, Institute of Clinical Psychology and Psychotherapy, Technische Universität Dresden, Germany” was involved in providing funding during the any aspect pertinent to the study. It also has been paying the salary of the corresponding author during the research process. We further acknowledge support from the Open Access Publication Fund of the TU Dresden and the Saxon State and University Library Dresden (SLUB).

## Conflicts of interest

The authors declare no conflicts of interest.
